# Combined Transcriptomic and Epitranscriptomic Profiling Identifies THBS1 as A Regulator of Enzalutamide Resistance in Prostate Cancer

**DOI:** 10.47248/chp2502020007

**Published:** 2025-04-21

**Authors:** Emmanuelle Hodara, Lisa Swartz, Aubree Mades, Daniel Bsteh, Tong Xu, Suhn K. Rhie, Amir Goldkorn

**Affiliations:** 1 Division of Medical Oncology, Department of Medicine, Keck School of Medicine of USC and Norris Comprehensive Cancer Center, Los Angeles, CA 90033, USA; 2 Department of Biochemistry and Molecular Medicine, Keck School of Medicine of USC, Los Angeles, CA 90033, USA

**Keywords:** epitranscriptomic1, m6A2, prostate cancer3, MeRIP-seq4, enzalutamide resistance5, drug resistance6, RNA methylation7

## Abstract

Cancer drug resistance arises not only from selection of resistant clones, but also through rapid activation of adaptive transcriptional programs. One mechanism of transcriptional regulation involves N6-methyladenosine (m^6^A) RNA modification, which dynamically regulates mRNA processing and alternative splicing, ultimately impacting cell fate and differentiation. In prostate cancer (PC), resistance to systemic therapies such as the androgen receptor pathway inhibitor (ARPI) enzalutamide is associated with a host of well-documented androgen receptor (AR) alterations, including amplification, mutation, and alternative splicing. Given these functions, we hypothesized that m^6^A modifications play a role in the transition to enzalutamide resistance in PC. To test this, we used methyl-RNA-immunoprecipitation followed by sequencing (MeRIP-seq) in parallel with RNA-seq to identify gene transcripts that were both differentially methylated and differentially expressed between enzalutamide-sensitive and enzalutamide-resistant PC cells. We filtered and prioritized these genes using clinical and functional database tools, including Gene Ontology (GO) enrichment analysis and Gene Set Enrichment Analysis (GSEA), The Cancer Genome Atlas (TCGA), and the Oncology Research Information Network (ORIEN) avatar. Using this approach, we identified 487 transcripts that were both differentially methylated and differentially expressed and validated six of the top 12 candidates via targeted qPCR and MeRIP-PCR. One of these, *THBS1*, was found to have increased m^6^A level associated with decreased transcript levels in enzalutamide-resistant cells, a finding recapitulated in publicly available preclinical and clinical data. Moreover, in enzalutamide-sensitive cells, depletion of *THBS1* by siRNA-knockdown induced resistance to enzalutamide. While *THBS1* has previously been implicated in aggressive PC phenotypes, we now show that *THBS1* downregulation directly contributes to a rapid transition to enzalutamide resistance, suggesting a novel role for this gene in PC hormonal therapy resistance. These results constitute the first comprehensive epitranscriptomic profiling of ARPI resistance and identify *THBS1* as a potential driver of acute resistance in prostate cancer.

## 1. Introduction

The backbone of systemic therapy for metastatic prostate cancer is androgen deprivation therapy (ADT), which reduces testicular androgen production, thereby reducing the activation of the Androgen Receptor (AR) and its downstream disease-driving signaling related to cell cycle and cancer progression [1]. Although patients initially respond well to treatment, they eventually progress to metastatic castrate-resistant prostate cancer (mCRPC) within 2–3 years of starting ADT [2,3]. After progression to mCRPC on ADT, second generation AR pathway inhibitors (ARPIs) such as enzalutamide and abiraterone can induce disease regression and prolong survival, but extend median survival only by 2–8 months due to additional acquired drug resistance mechanisms [3]. After progression on ARPIs, mCRPC is treated with various survival-prolonging chemotherapies or targeted agents but remains heavily driven by AR signaling via adaptive mechanisms in the form of amplification, mutation, overexpression and alternative splicing [4,5].

Epitranscriptomics, particularly N6-methyladenosine (m^6^A) RNA modification, is increasingly recognized as a driver of tumor plasticity and therapeutic resistance in various cancers, including prostate cancer. m^6^A plays a crucial role in regulating RNA stability, translation, and alternative splicing, contributing to dynamic changes in gene expression associated with cancer progression and drug resistance [6–10]. The regulation of m^6^A modifications involves three classes of proteins: writers, erasers, and readers. The m^6^A writer complex, comprising the highly conserved METTL3, METTL14, and WTAP (Wilms’ Tumor 1-Associating Protein), facilitates the deposition of m^6^A onto RNA [11]. Conversely, the demethylases ALKBH5 and FTO act as erasers, removing m^6^A modifications [12]. m^6^A readers, such as YTHDF1–3, recognize and selectively bind m^6^A-marked transcripts, mediating downstream effects [13]. This dynamic interplay between m^6^A deposition and removal is crucial for maintaining cellular functions and enabling tissue-specific gene expression. m^6^A modifications are considered master regulators of key signaling pathways, influencing mRNA stability, splicing, and epigenetic regulation [14,15]. While largely conserved, m^6^A patterns can vary by cell type, shaping tissue-specific regulatory pathways. Moreover, deregulation of m^6^A and its effectors has been implicated in cancer initiation, progression, drug resistance, and relapse [16].

Our group has recently shown that m^6^A modifications contribute to chemoresistance mechanisms in bladder cancer by altering transcript methylation patterns, impacting mRNA stability and expression, and modifying RNA degradation through differential interactions with m^6^A readers [17]. In prostate cancer, m^6^A modifications may play a similar role in shaping mechanisms of resistance to ARPIs. For example, one critical mechanism of resistance to ARPIs in prostate cancer involves the generation of AR splice variants. *ARV7*, the most extensively studied *AR* splice variant, lacks the ligand binding domain (LBD), is constitutively active and localizes to the nucleus independently of AR binding. ARV7 is overexpressed in resistant tumors, and its detection in circulating tumor cells (CTCs) is associated with resistance to ARPI therapy and decreased overall survival (OS) [18–22]. Although the precise molecular mechanism leading to this alternative splicing remains unclear, recent studies have demonstrated that the long non-coding RNA (lncRNA) *MALAT1* promotes expression of ARV7 and contributes to ARPI resistance, whereas *MALAT1* silencing suppresses PC progression by inhibiting AR signaling [23–26]. *MALAT1* exhibits high levels of m^6^A RNA modifications that contribute to a structural switch, wherein presence of the m^6^A modification increases likelihood of single-strandedness, structurally altering binding sites of RNA binding proteins [27]. Additionally, m^6^A plays a key role in regulating alternative splicing and splicing kinetics. Specifically, m^6^A modifications at splice junctions have been associated with fast, constitutive splicing, whereas intronic m^6^A modifications promote slow and alternative splicing [6,28]. These alterations enhance the ability of *MALAT1* to regulate alternative splicing and downstream oncogenic pathways.

Given these lines of evidence – that alternative splicing drives PC ARPI resistance and that m^6^A modification contributes to alternative splicing – we hypothesized that enzalutamide-resistant PC cells would have a m^6^A profile distinct from enzalutamide-sensitive PC cells, and that a subset of differentially methylated transcripts may play a role in resistance. To date, no studies have systematically mapped the epitranscriptomic landscape of APRI resistance in PC. Therefore, we set out to systematically identify changes in m^6^A RNA methylation and RNA expression between enzalutamide-sensitive *vs*. enzalutamide-resistant PC cells. Using unbiased transcriptome-wide m^6^A profiling and gene expression profiling followed by targeted validation, we found that enzalutamide resistance was associated with cancer relevant transcripts that were both differentially methylated and differentially expressed. One such transcripts, *THBS1* (antiangiogenic factor thrombospondin-1), was hypermethylated and downregulated upon enzalutamide treatment, and siRNA-mediated depletion of *THBS1* potentiated treatment resistance in enzalutamide-sensitive PC cells.

## 2. Results

### 2.1 Enzalutamide-sensitive and enzalutamide-resistant cells have distinct m6A profiles

**Figure 1.**
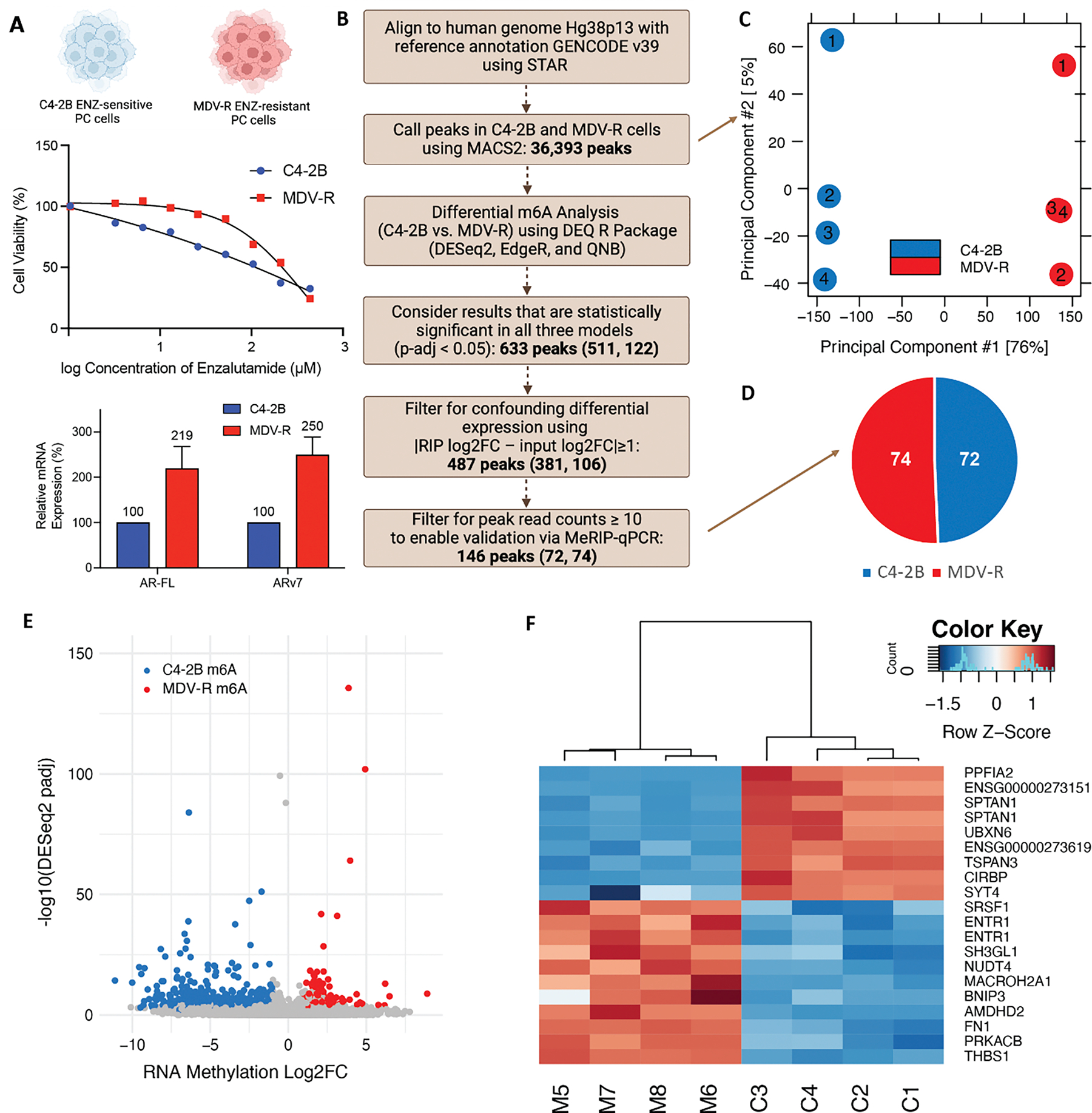
Enzalutamide-sensitive and Enzalutamide-resistant cells have distinct m^6^A profiles. (A) Profiling of C4–2B and MDV-R PC cells including cell viability curves with enzalutamide treatment and mRNA expression of *AR-FL* and *ARV7*. (B) MeRIP-seq informatics filtering pipeline for differential methylation analysis with summarized peak number for different filtering steps. Numbers in parentheses refer to: peaks hypermethylated in C4–2B, peaks hypermethylated in MDV-R. (C) PCA plot of MeRIP samples with 4 replicates per cell line using all 36,393 peaks. (D) Summary of filtering results comparing C4–2B to MDV-R, with 74 peaks hypermethylated in C4–2B and 72 peaks hypermethylated in MDV-R. (E) Volcano Plot of statistically significant differential MeRIP-results (p < 0.05 by DESEQ2, edgeR, and QNB). C4–2B m^6^A represents the 74 peaks hypermethylated in C4–2B cells with log2FC < −1 and MDV-R m^6^A represents the 72 peaks hypermethylated in MDV-R with log2FC > 1. (F) Heatmap of top 20 differentially methylated transcripts ranked by DESeq2padj. MDV-R replicates are labeled M5-M8. C4–2B replicates are labeled C1-C4.

We undertook a broad discovery approach comparing enzalutamide-sensitive C4–2B PC cells (IC_50_: 50uM enzalutamide) to established enzalutamide-resistant MDV-R PC cells (IC_50_: 200uM enzalutamide) that were derived from C4–2B by serial passage [29]. (Figure 1A). Resistant MDV-R cells have a 2.5-fold increase in ARV7 mRNA expression compared to sensitive C4–2B (Figure 1A). We used a low-input methyl-RNA-immunoprecipitation and sequencing (MeRIP-seq) protocol [30] and a stringent differential analysis pipeline incorporating all recommended best practices published to date [31], including 4 replicates per condition and filtering criteria to mitigate the confounding effect of differential expression on differential methylation (Figure 1B).

As a first pass quality control measure, we applied principal component analysis (PCA) to all 36,393 peaks called by MACS2 based on pull down *versus* input in all 8 replicates (4 sensitive and 4 resistant). The sensitive and resistant replicates clustered tightly into two distinct groups, with PC1 and PC2 representing 76% and 5% variation, respectively, reflecting the power of the study design to accurately discriminate the two conditions (Figure 1C). Encouraged by these preliminary results, we proceeded with differential methylation analysis using three statistical models (DESeq2, EdgeR and QNB) to evaluate statistical significance (p<0.05). We identified 633 differentially methylated peaks between enzalutamide-sensitive and resistant cells (Figure 1E), of which 487 remained after filtering for differential expression and 146 remained after selecting those with at least 10 reads. 74 of these peaks were hypermethylated in resistant cells, and 72 were hypermethylated in sensitive cells (Figure 1D). The volcano plot represents the 633 statistically significant peaks, highlighting the 487 peaks that meet all the filtering criteria and are unique to either sensitive or resistant cells (Figure 1E). The top 20 differentially methylated transcripts based on DESeq2 p-adj were visualized using a heatmap (Figure 1F).

### 2.2 Six cancer-relevant candidate transcripts were validated as differentially expressed and differentially methylated

**Figure 2.**
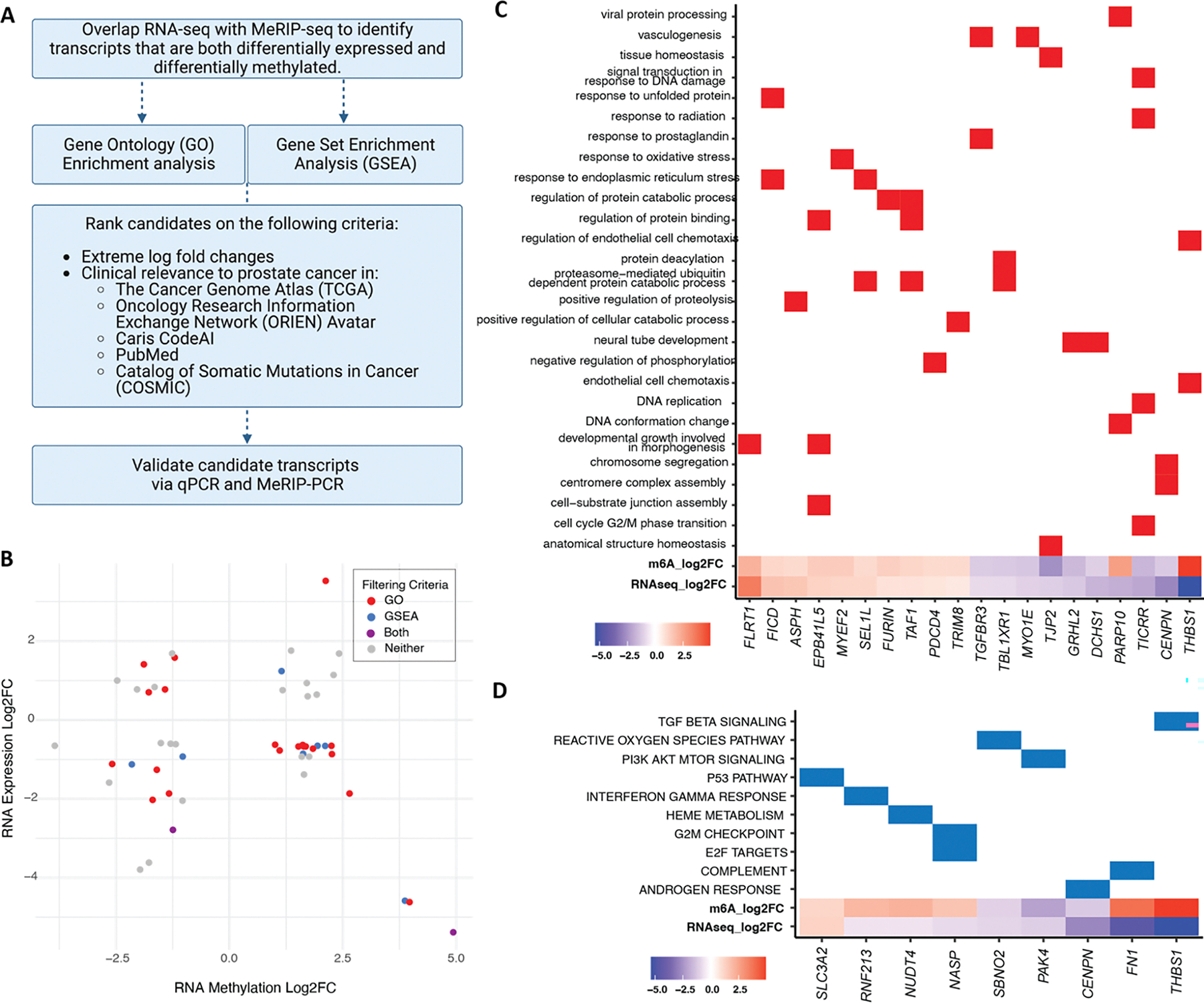
Identification of cancer-relevant transcripts that are both differentially methylated and differentially expressed in enzalutamide-resistant cells. (A) Discovery and filtering pipeline. (B) RNA expression Log2FC *vs*. RNA Methylation Log2FC of the 46 transcripts (52 peaks) that are both differentially methylated and differentially expressed between C4–2B and MDV-R. Peaks are colored based on filtering criteria: GO, GSEA, both or neither. (C) Differentially methylated and expressed transcripts statistically significantly associated with Gene Ontology (GO) Terms (p-adj <0.05). (D) Differentially methylated and expressed transcripts statistically significantly associated with Gene Set Enrichment Analysis (GSEA) Cancer Hallmarks (p-adj < 0.05).

**Figure 3.**
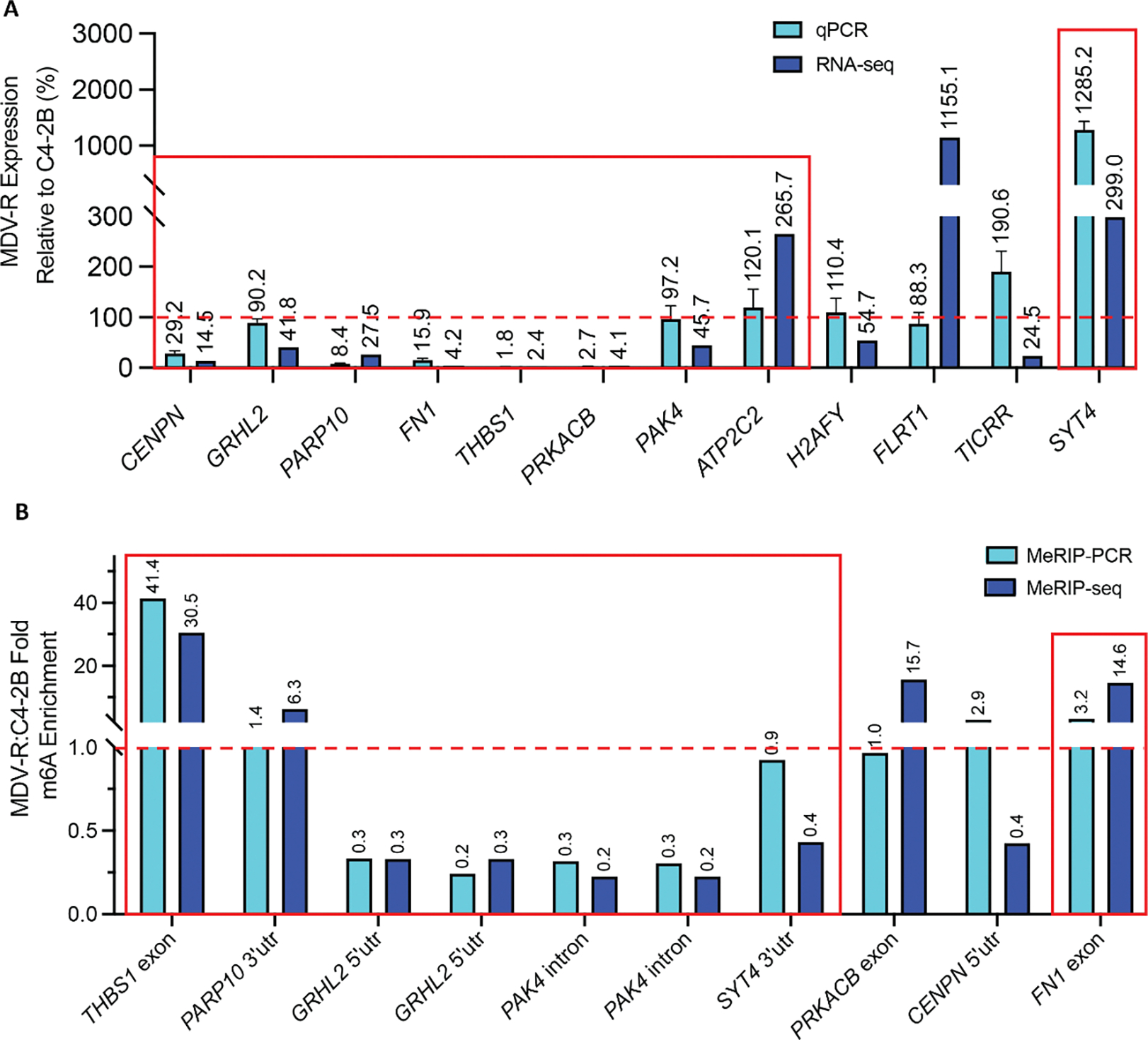
Validation of top 12 differentially methylated and expressed candidate transcripts. (A) 8 of 12 candidate transcripts (boxed in red) were validated as differentially expressed using qPCR, corroborating RNA-seq results. (B) 6 of the 8 candidate transcripts (boxed in red) were validated by MeRIP-qPCR, corroborating MeRIP-seq results. Red dotted lines in (A) and (B) represent the validation threshold.

To evaluate whether m^6^A alterations are associated with changes in gene expression, we overlapped the MeRIP-seq differential methylation results with RNA-seq differential expression from the same enzalutamide-sensitive and resistant cell lines (Figure S1A–C). We then applied the filtering pipeline outlined in Figure 2A in order to home in on transcripts relevant to cancer progression, as follows: We identified 46 candidate transcripts (52 out of 146 m^6^A peaks) that were both differentially expressed and differentially methylated (Figure 2B). We used Gene Ontology (GO) and Gene Set Enrichment Analysis (GSEA) to analyze the 46 transcripts. We identified 9 genes with membership in 10 GSEA Hallmarks of Cancer (p-adj<0.05, Figure 2C) and 20 genes with relevance to 27 GO terms like vasculogenesis and response to radiation (p-adj<0.05, Figure 2D). We further ranked the resulting transcripts based on log fold change and relevance to prostate cancer in existing literature and clinical databases such as The Cancer Genome Atlas (TCGA), the Oncology Research Information Exchange Network (ORIEN) Avatar and the Catalog of Somatic Mutations in Cancer (COSMIC). After ranking, we selected the top 12 transcripts for *in vitro* validation by qPCR and by targeted immunoprecipitation and PCR (MeRIP-PCR). 9 of the 12 differentially expressed transcripts by RNA-seq were also differentially expressed by qPCR (p<0.05), however only 8 were statistically significant (Figure 3A). Of the 8 transcripts (10 peaks) whose differential expression was validated by qPCR, 6 transcripts (8 peaks) were also validated as differentially methylated by MeRIP-qPCR (p<0.05, Figure 3B).

### 2.3 Depletion of *THBS1* induces resistance to enzalutamide

**Figure 4.**
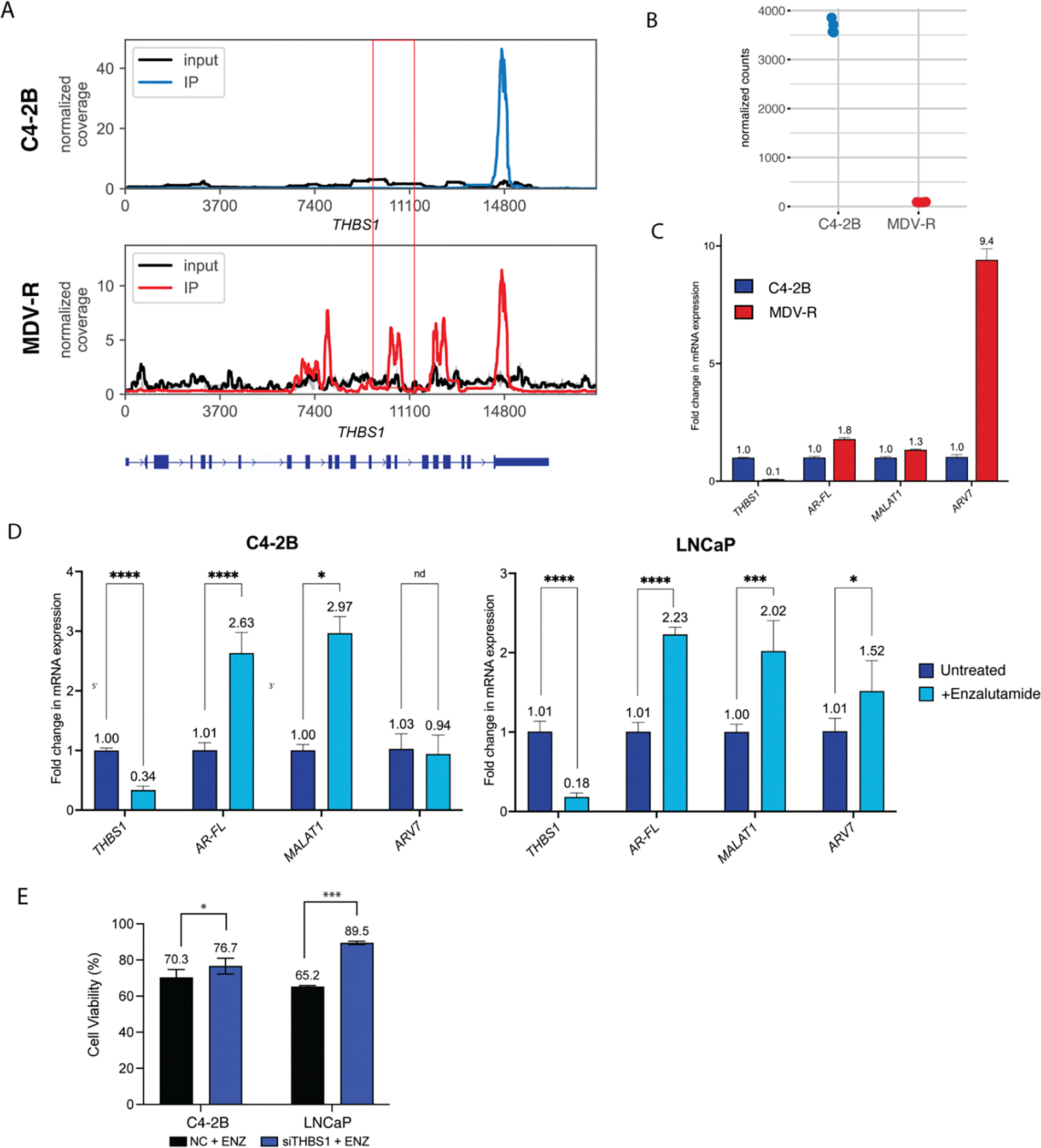
Validation of *THBS1* m^6^A methylation, mRNA expression, and effect of enzalutamide treatment. (A) M^6^A coverage plot for THBS1 in C4–2B and MDV-R cells depicting several exonic peaks, of which the peak at 9–10K (boxed) reached statistical significance after all normalizations and filtering and was validated by MeRIP-qPCR. 3’UTR peak is unchanged between the two cell lines. (B) Normalized RNA-seq count of *THBS1* in C4–2B and MDV-R cells. (C) RT-qPCR analysis of gene expression in enzalutamide-sensitive C4–2B and enzalutamide-resistant MDV-R PC cell lines. MDV-R cells show reduced *THBS1* expression and elevated *ARV7*, *AR-FL*, and *MALAT1* levels compared to C4–2B. Gene expression levels were normalized to the housekeeping gene *GAPDH* and further normalized to the expression levels in C4–2B cells. Graph displays mean ± SEM values in n=3 replicates. (D) RT-qPCR analysis of gene expression in C4–2B and LNCaP cells untreated or treated with short-term 5uM enzalutamide(ENZ) treatment. ENZ reduced *THBS1* expression and increased *AR-FL* and *MALAT1* levels in both cell lines. Data normalized to *GAPDH* and untreated controls. Graph displays mean ± SEM values in n=3 replicates; p values comparing treated cells to respective untreated cells. Significant codes: ‘****’: p<0.0001, ‘***’: p<0.001. (E) Functional validation of *THBS1* evaluating its effect on enzalutamide resistance via *siTHBS1* knockdown followed by 5uM enzalutamide treatment in C4–2B cells (CDI: 1.30) and LNCaP cells (CDI: 1.09). CDI < 0.7 indicates statistically significant synergy. CDI > 1.0 indicates statistically significant antagonism. Experiments were performed using three biological replicates. Significant codes: ‘*’: p< 0.05, ‘***’: p<0.001.

We determined if any of the 6 differentially methylated and expressed genes identified in enzalutamide-resistant MDV-R cells also played a role in the transition to treatment resistance in enzalutamide-sensitive cell lines. To do this, each of the 6 genes (GRHL2, THBS1, FN1, PARP10, PAK4, SYT4) was siRNA-depleted in enzalutamide-sensitive cell lines (Table S1). Subsequently, the cells were treated with enzalutamide and assessed for viability after 72 hrs. One candidate transcript, *THBS1*, was functionally validated in this manner. Analysis of the MeRIP-seq data revealed several exonic peaks in *THBS1* in MDV-R only, of which the peak at 9–10 K (boxed) reached statistical significance after all normalizations described above and was validated by MeRIP-PCR. Another peak at 3’UTR was not statistically different between enzalutamide-sensitive and enzalutamide-resistant cells (Figure 4A). Analysis of the RNA-seq data showed that *THBS1* mRNA expression was decreased 41-fold in enzalutamide-resistant cells (p-adj<E-100, Figure 4B). TCGA analysis comparing *THBS1* expression in prostate tumor and normal prostate samples showed significantly lower *THBS1* expression in tumor samples (Figure S2). The reduction in THBS1 mRNA and protein levels in MDV-R enzalutamide-resistant cells relative to C4–2B enzalutamide-sensitive cells was further confirmed by RT-qPCR, which showed a 92% reduction (Figure 4C), and western blot (Figure S3). MDV-R enzalutamide-resistant cells also had higher mRNA levels of *ARV7* (9.2-fold), *AR-FL* (1.8-fold), and *MALAT1* (1.3-fold) compared to C4–2B enzalutamide-sensitive cells (Figure 4C). Short-term enzalutamide treatment of enzalutamide-sensitive C4–2B and LNCaP PC cells led to a significant reduction in *THBS1* transcript levels, with a 66% and 82% decreases in C4–2B and LNCaP cells, respectively, and significantly increased expression of *AR-FL* (2.6-fold in C4–2B; 2.2-fold in LNCaP) and *MALAT1* (3.0-fold in C4–2B; 2.0-fold in LNCaP), however, *ARV7* was only upregulated in LNCaP cells (Figure 4D). *THBS1* depletion by siRNA knockdown significantly increased relative enzalutamide resistance in the two enzalutamide-sensitive PC cell lines, C4–2B and LNCaP, but was not sufficient to recapitulate the increases in *AR, ARV7*, or *MALAT1* seen with enzalutamide treatment (Figure 4C, Figure S4,S5).

**Figure 5.**
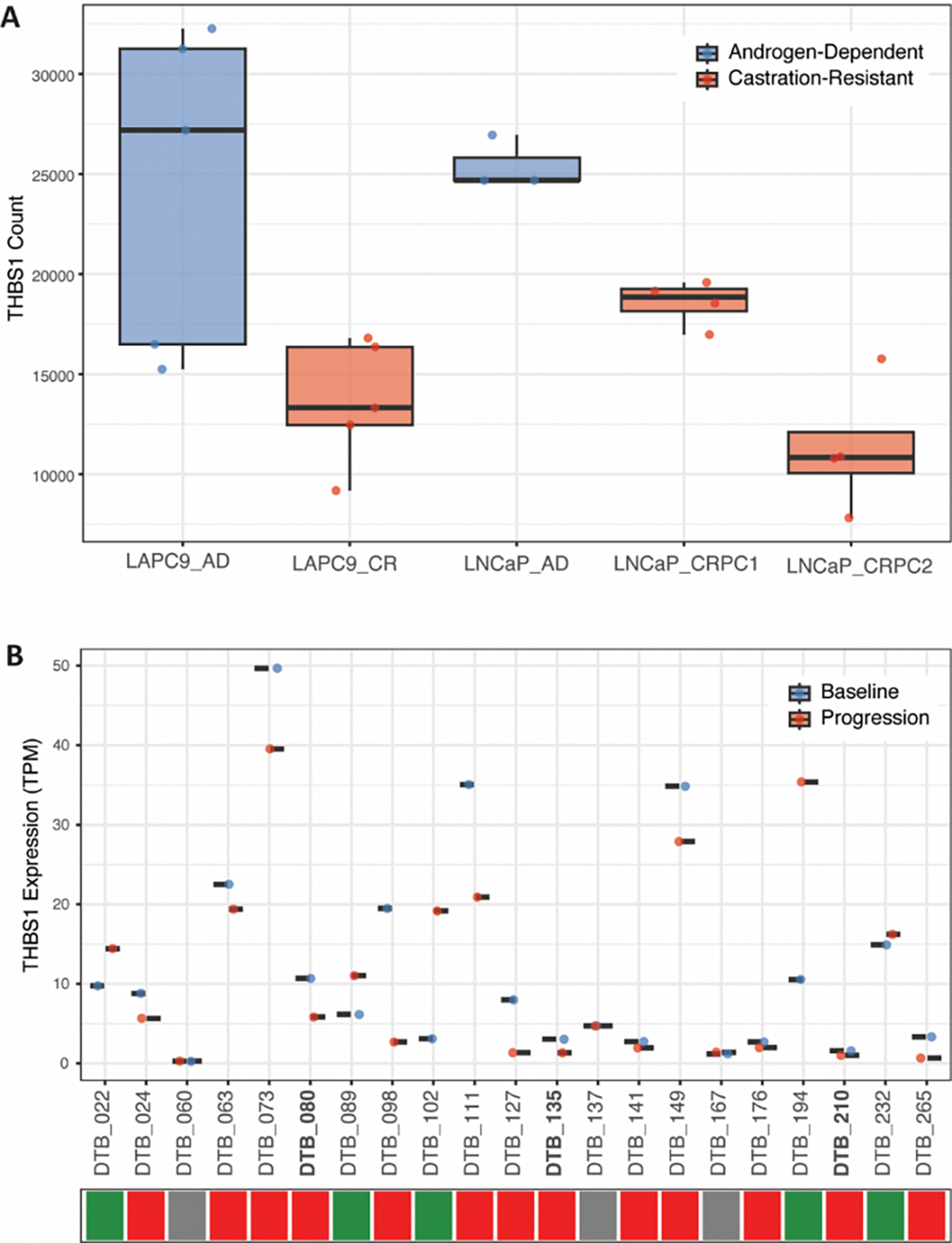
THBS1 Expression in samples from existing preclinical and clinical datasets. (A) THBS1 expression (counts) decreased in androgen-dependent (AD) xenografted LAPC9 and LNCaP PC cell lines after surgical castration of the host (PMID: 30190514)[32]. LNCaP_CRPC1 refer to the cell lines derived after surgical castration; LNCaP_CRPC2 refer to the castration-derived cell line after treatment with enzalutamide. P-values presented are from Wilcoxon rank sum test for LAPC9_AD *vs* LAPC9_CR (p=0.0556), LNCaP_AD *vs*. LNCaP_CRPC1 (p=0.0571), LNCaP_AD *vs*. LNCaP_CRPC2 (p=0.0571) LNCaP_CRPC1 *vs*. LNCaP_CRPC2 (p=0.0286). One outlier was removed from LNCaP_AD replicates based on Dixon-Q test. (B) THBS1 expression (TPM) at baseline and progression following enzalutamide treatment in 21 matched patient samples from exisiting clinical dataset (PMID: 36109521)[33]. Bolded patient samples (DTB_080, DTB_135, DTB_210) refer to patient samples termed “converters” having undergone enzalutamide-induced lineage plasticity as defined in the original paper. Colored-tiles refer to direction of THBS1 expression change from baseline to progression: increasing (green), decreasing (red), no change (grey).

We evaluated THBS1 differential expression from read counts data from previously published preclinical datasets [32] comparing androgen-dependent xenografted LAPC9 and LnCAP PC cell lines before and after surgical castration of the host (Figure 5A and Figure S6). Indeed, we saw a consistent trend of decreased THBS1 expression in castration-resistant cell lines compared to androgen-dependent cell lines. In particular, we observed a statistically significant (p=0.0286) decrease in THBS1 expression between the two castration-resistant LNCaP cell lines, LNCaP_PRC1 and LNCaP_PRC2, the latter of which was derived from the first following enzalutamide treatment. Finally, we compared THBS1 expression from a previously published clinical dataset [33] comparing 21 matched samples from PC patients at baseline and following progression on enzalutamide. 13 of the 21 matched pairs had a decrease in THBS1 expression (Figure 5B). Per previous analysis, three of the 21 matched pairs were deemed “converters” demonstrating lineage plasticity following enzalutamide treatment. Interestingly, all three of these converters (bolded) had a decrease in THBS1 expression, with log_2_ fold change of −2.12 and p-adj= 0.011 from previously published analysis [33].

## 3. Discussion

Prostate cancer (PC) resistance to androgen receptor pathway inhibitors (ARPIs) like enzalutamide remains a significant clinical challenge, often driven by adaptive mechanisms such as metabolic shifts, alternative splicing, and epigenetic modifications. Recent evidence has highlighted the importance of m^6^A RNA modifications in regulating transcriptome plasticity and resistance across various cancers, including prostate cancer, as they are known to influence processes such as RNA stability, alternative splicing, and translation, potentially contributing to therapy resistance. In this study we demonstrate that enzalutamide-resistant PC cells have a distinct m^6^A profile from enzalutamide-sensitive PC cells, raising the possibility that m^6^A modifications of certain genes may contribute to enzalutamide resistance. To identify gene candidates, we utilized a robust discovery and validation workflow that we previously published in a bladder cancer model of chemoresistance [17], and applied it to an established cell line model of enzalutamide resistance in prostate cancer [29]. We analyzed transcriptome-wide changes in m^6^A RNA modifications and gene expression using MeRIP-seq and RNA-seq, respectively, and identified 46 transcripts that were both differentially methylated and differentially expressed. We further filtered these 46 transcripts based on membership in GSEA Hallmarks of Cancer pathways including androgen response, TGF-β signaling, and relevance to GO terms like response vasculogenesis, endothelial cell chemotaxia, and response to radiation therapy. One of the candidate transcripts validated by MeRIP-PCR and qPCR was Thrombospondin-1 (*THBS1*), which was found to be hypermethylated and downregulated in resistant PC cells. Hence, *THBS1* becomes repressed as prostate cancer cells transition to resistant phenotype. *THBS1*, also known as *TSP1*, is a well-characterized adhesive glycoprotein that mediates cell-to-cell and cell-to-matrix interactions and is considered an antiangiogenic factor [34]. Consistent with our findings, THBS1 is downregulated in multiple human cancers including melanoma and breast cancer [35]. In prostate cancer, THBS1 was previously reported to be epigenetically repressed via the EZH2 axis with enzalutamide treatment, potentiating aggressive neuroendocrine transdifferentiation [36,37]. Notably, a recent study linked m^6^A to *THBS1* downregulation in prostate cancer, showing that it is a downstream target of the m^6^A writer co-factor METTL14, with methylation resulting in YTHDF2-mediated *THBS1* mRNA degradation and downregulation [38]. This raises the possibility that m^6^A-mediated *THBS1* transcript degradation may directly regulate this gene in response to enzalutamide treatment, a focus of ongoing work. Indeed, in our current study, short-term enzalutamide treatment of sensitive PC cell lines induced a rapid marked reduction in *THBS1* transcript levels, and *THBS1* knockdown potentiated an acute shift to enzalutamide resistance. This recapitulated the associations we first observed in established resistant MDV-R cells *vs*. sensitive C4–2B cells and assigned a direct role for THBS1 in the transition to enzalutamide resistance. THBS1 knockdown significantly potentiated enzalutamide resistance in both LNCap and C4–2B cells, though its effect was more modest in the latter. While THBS1 knockdown efficiency was virtually identical between the two cell lines, C4–2B has a higher expression of THBS1 at baseline, resulting in higher absolute levels of THBS1 after knockdown. C4–2B is an androgen-independent metastatic derivative of LNCaP and may also be less affected by THBS1 suppression since it is already more aggressive, utilizing multiple compensatory pathways to support survival. This is further supported by the fact that, following enzalutamide treatment, THBS1 transcript levels fall more sharply in LNCaP (82%, 1.01 *vs*. 0.18) than in C4–2B (66%, 1.0 *vs*. 0.34) (Figure 4D), suggesting that THBS1 down regulation may be more critical for enzalutamide resistance in LNCaP than C4–2B.

Using existing preclinical [32] and clinical datasets [33], we also evaluated THBS1 differential expression between androgen-dependent and castration-resistant PC cells, and matched samples from PC patients at baseline and after progression on enzalutamide. We saw the same downtrend in THBS1 expression in a majority of the patient samples following progression on enzalutamide, corroborating our observations and highlighting their translational potential. In particular, three patients deemed “converters” whose tumors had undergone lineage plasticity in response to enzalutamide treatment were observed to have significant THBS1 down regulation in that study [33].

Acute and chronic enzalutamide exposure also led to significantly increased levels of known drivers of ARPI resistance, including *ARV7*, a constitutively active splice variant of the androgen receptor, *AR-FL* (androgen receptor full length), and *MALAT1*, a lncRNA known to promote ARV7 expression [16–24]. Therefore, we investigated whether *THBS1* depletion alone without enzalutamide treatment would be sufficient to upregulate these resistance-associated genes. This did not occur, suggesting that *THBS1* depletion alone is not sufficient to upregulate these genes, or, alternatively, that *THBS1*’s role in enzalutamide resistance is mediated through alternate mechanisms.

To our knowledge, this is the first comprehensive epitranscriptomic data set analysis of enzalutamide resistance in PC, which highlights the distinct epitranscriptomic signatures of enzalutamide-resistant PC cells in addition to identifying several differentially methylated and expressed gene transcripts that may merit further investigation. In particular, it identifies a novel role for *THBS1* as a regulator of acute enzalutamide resistance. Although the specific mechanisms linking enzalutamide exposure to *THBS1* depletion have yet to be elucidated, our findings of increased transcript methylation suggest this may be one potential mechanism, as observed previously in PC progression [39]. Additional mechanistic studies on enzalutamide-mediated *THBS1* depletion and its downstream targets, including rescue overexpression of THBS1 in resistant cell lines and *in vivo* validation, may offer new mechanistic insights and therapeutic opportunities to reverse ARPI resistance.

## 4. Materials & Methods

### 4.1 Cell culture

Human prostate cancer cell lines, C4–2B and LNCaP, were cultured in RPMI 1640 and DMEM (Mediatech Inc., Manassas, VA), respectively, supplemented with 10% heat-inactivated fetal bovine serum (Omega) and 1% penicillin/streptomycin (100 units/mL, Invitrogen), at 37 °C and 5% CO_2_. Prior to conducting all experiments, the cell lines were authenticated using 9-marker short tandem repeat (STR) profiling and testing interspecies and mycoplasma contamination (CellCheck 9 Plus, IDEXX BioAnalytics, Columbia, MO). We maintain stringent, good cell culture practice (GCCP), and keep extensive records of cell lines’ culture and harvesting condition, including passage count and cell density. We performed all experiments below passage 20, after which cultures were restarted using thawed cells from earlier passages. MDV-R cells were generated by serial desensitization of C4–2B cells with enzalutamide and were a generous gift from the Gao Lab (UC Davis) [29]. MDV-R cell lines were maintained in RPMI 1640 media with 5μM enzalutamide (Catalog#MDV3100, SelleckChem, Houston, TX).

### 4.2 RNA isolation

Total RNA was extracted from cell lines using Trizol reagent and Direct-zol RNA extraction kit (R2071, Zymo Research), which included treatment with DNase I for 20min at 37 °C. Total RNA was extracted from organoids using RNEasy Micro Kit (Catalog# 74004, Qiagen, Germany). The concentration of total RNA was measured by Qubit RNA HS Assay Kit (Catalog# Q32855, Thermo Fisher Scientific) or via nanodrop, depending on the starting amount.

### 4.3 RNA fragmentation and MeRIP

MeRIP was performed on RNA chemically fragmented to ~100nt fragments as previously described [17,30]. Briefly, 3–5 μg (18μL volume) of purified RNA was incubated with 2μL 10X RNA Fragmentation Buffer (100 mM Tris-HCl, 100 mM ZnCl_2_ in nuclease free H_2_O) in a preheated thermal cycler for 4 ~ 5 min at 70 °C. The reaction was stopped with 0.5M 2μL EDTA, followed by 178μL of H_2_O, 20μL of sodium acetate (3 M, pH 5.2, S7899, Sigma-Aldrich, St. Louis, MO), 14.4μL of glycogen (5 mg/ml, Catalog#AM9510, Thermo Fisher Scientific) and 500μL of 100% ethanol and incubated at −80 °C overnight. Fragmented RNA was pelleted (centrifuged 30min at 12,000g at 4 °C), washed once with 75% ethanol and resuspended in RNAse-free water (10 μL H_2_O per 1 μg human total RNA). Size distribution was assessed using RNA 6000 Pico Kit on BioAnalyzer (Catalog# 50671513, Agilent Technologies, Santa Clara, CA).

30 μL of protein-A magnetic beads (Catalog# 10002D, Thermo Fisher Scientific) and 30 μL of Protein-G magnetic beads (Catalog# 10004D, Thermo Fisher Scientific) were washed twice with IP buffer (150 mM NaCl, 10 mM Tris-HCl, pH 7.5, 0.1% IGEPAL CA-630 in nuclease free H_2_O), and incubated with 5μg anti-m^6^A antibody (Catalog# E1610, NEB, Ipswich, MA) at 4 °C overnight. The bead-antibody mixture was washed twice with IP buffer and resuspended in 500 μL IP buffer containing the fragmented RNA, 100 μL of 5X IP buffer and 5 μL RNasin Plus RNAse Inhibitor (Catalog# N2611, Promega, Madison, WI), and incubated for 2 hours at 4 °C.

The RNA reaction mixture was washed twice with 1000μL IP buffer, twice with 1000 μL low-salt IP buffer (50 mM NaCl, 10 mM Tris-HCl, pH 7.5, 0.1% IGEPAL CA-630 in nuclease free H_2_O), and twice with 1000μL high-salt IP buffer (500 mM NaCl, 10 mM Tris-HCl, pH 7.5, 0.1% IGEPAL CA-630 in nuclease free H_2_O) for 10min each at 4 °C. After the washes, the m^6^A-containing fragments was eluted from the beads in 200 μL of RLT Buffer supplied in the RNeasy Micro Kit (Catalog# 74004, Qiagen, Germany) for 2 min at room temperature. Using magnetic separation rack (Catalog# 1231D, Thermo Fisher Scientific) to pull beads to the side of the tube, supernatant was collected and combined with 400 μL 100% ethanol. The mixture was transferred to an RNeasy MicroElute spin column (RNeasy Micro Kit) and centrifuged at >12,000rpm at 4 °C for 1 min. The column membrane was washed with 500μL RPE Buffer (RNeasy Micro Kit) once, and with 500 μL 80% ethanol once. The column was centrifuged at full speed for 5min at 4 °C to remove residual ethanol. The m^6^A-containing fragments were eluted with 14 μL nuclease-free water.

### 4.4 RT-qPCR and MeRIP-qPCR

cDNA synthesis was performed using qScript cDNA SuperMix (Catalog# 95408–500, QuantaBio, Beverly, MA). Real Time PCR was performed using PerfeCTa SYBR Green FastMix (Catalog# 95071–250, QuantaBio) using a BioRad CFX96 Real Time PCR Detection System. Primer sequences can be found in Table S2. All experiments were performed in biological triplicates, and additional technical triplicates were used for all RT-qPCR experiments.

### 4.5 RNA-seq

RNA-seq libraries were prepared using NEBNExt Ultra II RNA Library Prep Kit for Illumina (Catalog# E7420, NEB) according to manufacturer’s protocol. RNA-seq reads were aligned to human genome hg38 with reference annotation GENCODE v39 and counted using STAR (version 2.7.0) [39,40]. Only uniquely mapped reads without duplicates were selected using samtools (version 1.10) [41]. Read counts were assigned to genes using Subread featureCounts [42]. Read counts were normalized using DESeq2 package in R (version 4.1.3). To generate more accurate log2fold change estimates, shrinkage of the LFC estimates towards zero was applied using DESeq2 [43]. Differentially expressed transcripts with absolute |log2FC| > 0.5 and adjusted-p value < 0.05 were retained. Further filtering (|log2FC| > 0.5, FDR<0.00001) was later performed for gene selection for downstream qPCR validation. Gene set enrichment analysis and Gene Ontology analysis were implemented and visualized using clusterProfiler package [44–46].

### 4.6 Differential MeRIP-seq analysis

MeRIP libraries were prepared using SMARTer Stranded Total RNA-Seq Kit v2-Pico Input Mammalian (Catalog # 634413). MeRIP-seq reads were aligned to human genome hg38 with reference annotation GENCODE v39, and counted using STAR (version 2.7.0) [39,40]. Only uniquely mapped reads without duplicates were selected using samtools (version 1.10)[41]. IP over input peaks were called using MACS2 callpeak using the parameters “-nomodel -extsize100 -gsize300e6” [47]. Differential m^6^A analysis was performed using DEQ package in R as previously published [31]. Briefly, DEQ runs statistical analysis using DESeq2, edgeR and QNB packages [31,43,48,49]. Results that were statistically significant with adjusted p-value < 0.05 using all three packages were considered significant. Gene and peak expression changes were estimated as log2 fold changes from DESeq2. Additional filtering to mitigate the confounding effect of differential gene expression on determining differential methylation was applied using |peak IP log2FC- gene input log2FC| ≥ 1. Peaks with fewer than 10 read counts were also removed to mitigate the confounding effect of differential gene expression.

### 4.7 siRNA Knockdown

Dicer-substrate short interfering RNA (DsiRNAs) targeting transcripts of interest and corresponding negative control were purchased from Integrated DNA Technologies (IDT, Coralville, IA). DsiRNA sequences, concentrations and timepoints used can be found in Table S3. C4–2B and LNCaP cells, transfection was performed 24 hours after seeding. DharmaFECT #3 (Catalog# T-2003–02, Horizon Discovery, Waterbeach, UK) and OPTI-MEM I reagents (Catalog# 31985062, ThermoFisher) were used to transfect cells at 70% confluence according to manufacturer’s protocol. Cells were harvested and total RNA was extracted at 24 or 48 hours depending on the transcript. Knockdown efficiency was evaluated by RT-qPCR. In subsequent experiments, total RNA was extracted at 48 or 72 hours post-transfection followed by RT-qPCR of the downstream target genes.

### 4.8 Enzalutamide resistance assay

C4–2B or LNCaP cells were seeded and transfected the following day with corresponding siRNA or negative control for 24–48 hours prior to treating reseeding cells with 5uM enzalutamide (Catalog# S1166, Selleck Chemicals) for 72 hours. Enzalutamide was dissolved in DMSO and stored in single use aliquots at −80 °C. MTS proliferation assay was performed using CellTiter 96 AQueous One Solution Cell Proliferation Assay (Catalog#G3582, Promega) according to manufacturer’s protocol. Cell viability was calculated using: Percentage Viability= (Absorbance[Sample]/Absorbance[NC − ENZ])×100%. Coefficient of drug interaction (CDI) to evaluate synergy was calculated using: CDI= Percentage Viability[Condition + ENZ]/(Percentage Viability [Condition − ENZ] x Percentage Viability [NC – ENZ]). CDI > 1.0 indicates antagonism and CDI < 0.7 indicates significant synergy [50]. Experiments were repeated in 3–4 biological replicates, using 6 technical replicates.

### 4.9 THBS1 Expression Analysis in Preclinical and Clinical Datasets

Using previously published preclinical dataset (PMID: 30190514) comparing androgen-dependent (AD) and castration-resistant (CR) LAPC9 and LNCaP PC xenografted cell lines [32]. Briefly, AD xenograft tumors were routinely maintained in intact mice; while CR lines LAPC9_CR and LNCaP_CRPC1) were established by serially passaging AD tumor cells in surgically castrated mice. CR tumors that became enzalutamide-resistant following enzalutamide treatment were deemed secondary CRPC (LNCaP_CRPC2).Using read count data from 4–5 replicates for each cell line, DeSEQ2 was used to evaluate differential expression of THBS1 between the samples. Dixon-Q test was used to identify outliers. Analysis was repeated after removal of one outlier replicate. Both analyses (with and without outliers) are presented in Supp. Figure 5A and Figure 5A, respectively. Wilcoxon rank sum test was used to calculate p-values.

Using previously published clinical dataset (PMID: 36109521) [33] comparing 21 matched pairs of prostate cancer patient samples at baseline and at progression on enzalutamide. Using TPM values, we evaluated THBS1 differential expression between matched samples. Three of the matched pairs were deemed “converters” meaning they underwent lineage plasticity following enzalutamide treatment.

### 4.10 Western blot

Whole cell lysates were extracted from PC cells using RIPA lysis buffer (Sigma-Aldrich, Cat# R0278). Total protein concentration was determined by Lowry Assay using the BCA Protein Assay Kit (Bio-Rad, Hercules, CA, USA). 100 μg protein lysate samples were boiled in loading buffer for 10 minutes before running on 3–8% Tris-Acetate gradient gels (Invitrogen, Cat# EA0375BOX) with PageRuler Plus Prestained Protein Ladder (10–250 kDa) (ThermoFisher, Catalog#26619). Western blot transfer was performed using the iBlot2 Dry Blotting System (ThermoFisher Scientific) and PVDF mini iBlot 2 Transfer Stack (Invitrogen, Catalog #IB24002). Membranes were then blocked in Odyssey blocking buffer (LI-COR, Lincoln, NE, USA) and incubated with primary antibodies overnight at 4 °C. Membranes were then washed 3 times in 1X TBST + 0.10% Tween20 Buffer, and then incubated in respective secondary anti-mouse (goat anti-mouse IRDye 680RD, 1:5000, LI-COR, Cat# 926–68070) and secondary anti-rabbit (goat anti-rabbit IRDye 800CW, 1:5000, LI-COR, Cat# 926–68070) for 45 minutes at room temperature. After 3 more washes in 1X TBST + 0.10% Tween20 Buffer, membranes were visualized using an Odyssey DLx Imaging System (LI-COR). The primary antibodies used were as follows: GAPDH (Cat#437000, 1:5000; Invitrogen), THBS1 (Cat#37879, 1:1000; Cell Signaling Technology).

### 4.11 Statistical Analysis

P-values were calculated using two-tailed Student’s t-test or Wilcoxon rank sum testbetween two groups, and Wilcoxon signed rank test between matched groups. Data are presented as means +/− SEMs from at least three independent experiments. Significant codes: ‘****’: p< 0.00001, ‘***’: p< 0.001, ‘**’: p<0.01, ‘*’: p <0.05, ‘.’: p<0.1, and ‘ns’: not significant GraphPad Prism version 9, Excel version 16.68, or R version 4.1.3 were used for statistical analysis.

## Supplementary Material

Supplementary Material Figure S1. Differential RNA-seq between C4–2B and MDV-R PC cells. (A) PCA plot of RNA-seq samples with 4 replicates per cell line. (B) Volcano Plot of statistically significant differential RNA-seq results (p-adj<0.00001). Downregulated represents the transcripts with log2FC < −0.5 and Upregulated with log2FC > 0.5. (C) Heatmap of top 20 differentially expressed transcripts ranked by log2FC. MDV-R replicates are labeled M5-M8. C4–2B replicates are labeled C1-C4.
Figure S2. THBS1 expression in normal tissue vs. prostate cancer tissue in PC patients from TCGA. Significance code: **** p < 0.00001.
Figure S3. THBS1 protein expression between MDV-R and C4–2B PC cells. Western blot for THBS1 and GAPDH in MDV-R and C4–2B cells.
Figure S4. siRNA-knockdown efficiency and enzalutamide viability in PC cells. *THBS1* siRNA-knockdown efficiency for (A) C4–2B and (B) LNCaP cells. NC= negative control (scrambled siRNA). (C) LNCaP cell viability after 72 hours of enzalutamide treatment at varying concentration.
Figure S5. Effect of acute THBS1 depletion on *AR-FL*, *MALAT1*, and *ARV7* expression in PC cells. *THBS1* siRNA-knockdown efficiency for (A) C4–2B and (B) LNCaP cells. NC= negative control (scrambled siRNA). Fold change in mRNA expression of *AR-FL*, *MALAT1* and *ARV7* in (C) C4–2B and (D) LNCaP cells.
Figure S6. THBS1 Expression in Preclinical datasets before removing outlier from LNCaP_AD replicates. In existing preclinical dataset (PMID: 30190514), THBS1 expression (counts) decreased in androgen-dependent (AD) xenografted LAPC9 and LNCaP PC cell lines after surgical castration of the host. LNCaP_CRPC1 refer to the cell lines derived after surgical castration; LNCaP_CRPC2 refer to the castration-derived cell line after treatment with enzalutamide. P-values presented are from Wilcoxon rank sum test for LAPC9_AD vs LAPC9_CR (p=0.0556), LNCaP_AD vs. LNCaP_CRPC1 (p=0.3429), LNCaP_AD vs. LNCaP_CRPC2 (p=0.2) LNCaP_CRPC1 vs. LNCaP_CRPC2 (p=0.0286).
Table S1. Summary table of functional validation for the 6 transcripts previously validated by qPCR and MeRIP-qPCR.
Table S2. RT-qPCR and MeRIP-qPCR primers for prostate cancer targets.
Table S3. siRNA references and conditions for prostate cancer targets.

## Data Availability

The datasets generated for this study can be found in the GEO Database: GSE249750. All other relevant data are within the manuscript. Link: https://urldefense.com/v3/__https://www.ncbi.nlm.nih.gov/geo/query/acc.cgi?acc=GSE249750__;!!LIr3w8kk_Xxm!rDJbDcCVPWv-VrddOabaP8rFUqPTHQ_N64hIA5-G26mP-PclaTKEkNtJp1cFShgZrLi0zfX52Ne1g_VXUQ$ Reviewer Token: cbsbicwidxqftkr
