# Supplementary material for: Combined Transcriptomic and Epitranscriptomic Profiling Identifies THBS1 as A Regulator of Enzalutamide Resistance in Prostate Cancer
Source: Cancer Heterog Plast. Author manuscript; Available in PMC 2025 Apr 29. (PMC12040338; doi:10.47248/chp2502020007)
Supplement: Supplementary Material — Figure S1. Differential RNA-seq between C4–2B and MDV-R PC cells. (A) PCA plot of RNA-seq samples with 4 replicates per cell line. (B) Volcano Plot of statistically significant differential RNA-seq results (p-adj<0.00001). Downregulated represents the transcripts with log2FC < −0.5 and Upregulated with log2FC > 0.5. (C) Heatmap of top 20 differentially expressed transcripts ranked by log2FC. MDV-R replicates are labeled M5-M8. C4–2B replicates are labeled C1-C4. Figure S2. THBS1 expression in normal tissue vs. prostate cancer tissue in PC patients from TCGA. Significance code: **** p < 0.00001. Figure S3. THBS1 protein expression between MDV-R and C4–2B PC cells. Western blot for THBS1 and GAPDH in MDV-R and C4–2B cells. Figure S4. siRNA-knockdown efficiency and enzalutamide viability in PC cells. THBS1 siRNA-knockdown efficiency for (A) C4–2B and (B) LNCaP cells. NC= negative control (scrambled siRNA). (C) LNCaP cell viability after 72 hours of enzalutamide treatment at varying concentration. Figure S5. Effect of acute THBS1 depletion on AR-FL, MALAT1, and ARV7 expression in PC cells. THBS1 siRNA-knockdown efficiency for (A) C4–2B and (B) LNCaP cells. NC= negative control (scrambled siRNA). Fold change in mRNA expression of AR-FL, MALAT1 and ARV7 in (C) C4–2B and (D) LNCaP cells. Figure S6. THBS1 Expression in Preclinical datasets before removing outlier from LNCaP_AD replicates. In existing preclinical dataset (PMID: 30190514), THBS1 expression (counts) decreased in androgen-dependent (AD) xenografted LAPC9 and LNCaP PC cell lines after surgical castration of the host. LNCaP_CRPC1 refer to the cell lines derived after surgical castration; LNCaP_CRPC2 refer to the castration-derived cell line after treatment with enzalutamide. P-values presented are from Wilcoxon rank sum test for LAPC9_AD vs LAPC9_CR (p=0.0556), LNCaP_AD vs. LNCaP_CRPC1 (p=0.3429), LNCaP_AD vs. LNCaP_CRPC2 (p=0.2) LNCaP_CRPC1 vs. LNCaP_CRPC2 (p=0.0286). Table S1. Summary table of functional valida [file NIHMS2076661-supplement-Supplementary_Material.pdf]

Cancer Heterogeneity and Plasticity
https://doi.org/10.47248/chp2502020007

Original Research

Combined Transcriptomic and Epitranscriptomic Profiling Identifies THBS1 as A Regulator of Enzalutamide Resistance in Prostate Cancer

Emmanuelle Hodara [1], Lisa Swartz [1], Aubree Mades [1], Daniel Bsteh [1], Tong Xu [1], Suhn K. Rhie [2], Amir Goldkorn [1,2,*]

1. Division of Medical Oncology, Department of Medicine, Keck School of Medicine of USC and Norris Comprehensive Cancer Center, Los Angeles, CA 90033, USA; Emails: emmanuelle.hodara@usc.edu (E.H.); lmswartz@usc.edu (L.S.); mades@usc.edu (A.M.); db_216@usc.edu (D.B.); Tong.Xu@med.usc.edu (T.X.)
2. Department of Biochemistry and Molecular Medicine, Keck School of Medicine of USC, Los Angeles, CA 90033, USA; Email: rhie@usc.edu

* **Correspondence:** Amir Goldkorn; Email: agoldkor@med.usc.edu

Supplementary Materials

Table S1. Summary table of functional validation for the 6 transcripts previously validated by qPCR and MeRIP-qPCR.

Target	Peak # Location	Differential Methylation	Differential Expression	Expected Effect of KD on cell death in enzalutamide sensitive cells	Actual Effect of KD on Resistance: CDI	Corroborates?
GRHL2	1: utr5	↓	↓	Antagonism	NA	NA
THBS1	1: exon	↑	↓	Antagonism	CDI: 1.09<br>Antagonism	**YES**
FN1	1: exon	↑	↓	Antagonism	CDI: 1.00<br>No Effect	NO
PARP10	1: utr3<br>1: exon	↑ ↓	↓	Antagonism	CDI: 0.94<br>No Effect	NO
PAK4	1: intron	↓	↓	Antagonism	CDI: 0.89<br>Synergy	NO
SYT4	1: utr3	↓	↑	Synergy	CDI: 0.83<br>Synergy	YES:<br>Not Significant

*Arrows indicate expression or methylation levels in MDV-R compared to C4-2B. CDI is a coefficient of synergy between siRNA knockdown and Enzalutamide treatment in enzalutamide-sensitive LnCAP cells. CDI < 0.7 indicates statistically significant synergy and CDI> 1 indicates statically significant antagonism. CDI is the average of 3 biological replicates. NA: *GRHL2* could not be knocked down successfully.

Table S2. RT-qPCR and MeRIP-qPCR primers for prostate cancer targets.

RT-qPCR Primers	FWD	REV
GAPDH	TCA AGG CTG AGA ACG GGA AG	GGA CTC CAC GAC GTA CTC AG
SETD7	GGGGTTCAGAGACCTGGAAT	GCATGGTGAGAGGATGTGAC
CLUC	GCTTCAACATCACCGTCATTG	CACAGAGGCCAGAGATCATTC
GLUC	CGACATTCCTGAGATTCCTGG	TTGAGCAGGTCAGAACACTG
CENPN	TGA ACT GAC AAC AAT CCT GAA GG	CTT GCA CGC TTT TCC TCA CAC
GRHL2	TCA ATA CCC GAA GAG CCT ACA	CTT GGC TGT CAC TTG CTT TGC
PARP10	CAG CTC TAC CAT GAG GAC CTT	CGA AAG CCA GTC ATA TCC GGT
FN1	CGG TGG CTG TCA GTC AAA G	AAA CCT CGG CTT CCT CCA TAA
THBS1	AGA CTC CGC ATC GCA AAG G	TCA CCA CGT TGT TGT CAA GGG
PRKCAB	TTC AGC AAC TCA CAG TTA AGC A	GGC ACA CTC ATA CTT CTG ACC
PAK4	GGA CAT CAA GAG CGA CTC GAT	CGA CCA GCG ACT TCC TTC G
ATP2C2	CCA GAG CGT TTT GTG TGG ACT	GGG GTT CTT AAA CTG ATC CAG G
H2AFY	CGG ATG CTG CGG TAC ATC AA	CTC CGC TGT CAG GTA TTC CAG
FLRT1	AGC GAG ATG GAC GAG TGT TTT	GGG TAG TCA ATG TTG GAG TCG
TICRR	AAG CTA TCA GCG ATC TCG GC	TGA ACC TGG CAA GGA GCA AA
SYT4	ATG GGA TAC CCT ACA CCC AAA T	TCC CGA GAG AGG AAT TAG AAC TT
AR-FL	AAC AGA AGT ACC TGT GCG CC	TTC AGA TTA CCA AGT TTC TTC AGC
MALAT1	GAA TTG CGT CAT TTA AAG CCT ACT T	GTT TCA TCC TAC CAC TCC CAA TTA AT
ARV7	AAC AGA AGT ACC TGT GCG CC	TCA GGG TCT GGT CAT TTT GA
**MeRIP-qPCR Primers**	**FWD**	**REV**
THBS1 exon m^{6}A region	CCC TGG CTT CTC ATA GCC AA	TGC TGA GCA AGT CCA GTA GC
PARP10 3’UTR m^{6}A region	GAG TCC AGG GTT TGA GGG AG	GTC ACT TGC TTC ACG GAG GT
GRHL2 5’UTR m^{6}A region 1	TCC CCT TTG GGC CTT GAT AG	GTA TTC ATC CAG GAG CGG GAG
GRHL2 5’UTR m^{6}A region 2	CCC TTA GGA ATG GTC TCA GCT C	ATC GGT GGG AGC AGC TAA AA
PAK4 intron m^{6}A region	CCT GCG TTA TGC TGG AGT GT	TGG CAT TTG GCA GGA GCT TA
SYT4 3’UTR m^{6}A region	TTA ACT TCT GGC TGC CGT GA	GTA TCA TGG GCC GTG GAA CA
PRKACB exon m^{6}A region	AGC CAA AGC CAA AGA AGA CT	CTG AGT TGG ATT CTC CCA TTT TT
CENPN 5’UTR m^{6}A region	ACT TTG TTG TGC TGT TTT TGT TTT G	TTG ATG AAC TCA GCA ACA GTC TC
FN1 exon m^{6}A region	CCA CTC ATC TCC AAC GGC AT	GGC TTG AAC CAA CCT ACG GA

Table S3. siRNA references and conditions for prostate cancer targets.

Target	Company	Catalog#	Concentration	Time
Negative Ctrl	IDT	51-01-14-03	10nM	24-48HRS
THBS1	IDT	hs.Ri.THBS1.13.3	25nM	48HRS
PARP10	IDT	hs.Ri.PARP10.13.1	25nM	48HRS
PAK4	IDT	hs.Ri.PAK4.13.3	25nM	48HRS
SYT4	IDT	hs.Ri.SYT4.13.3	25nM	48HRS
FN1	IDT	hs.Ri.FN1.13.1	25nM	24HRS

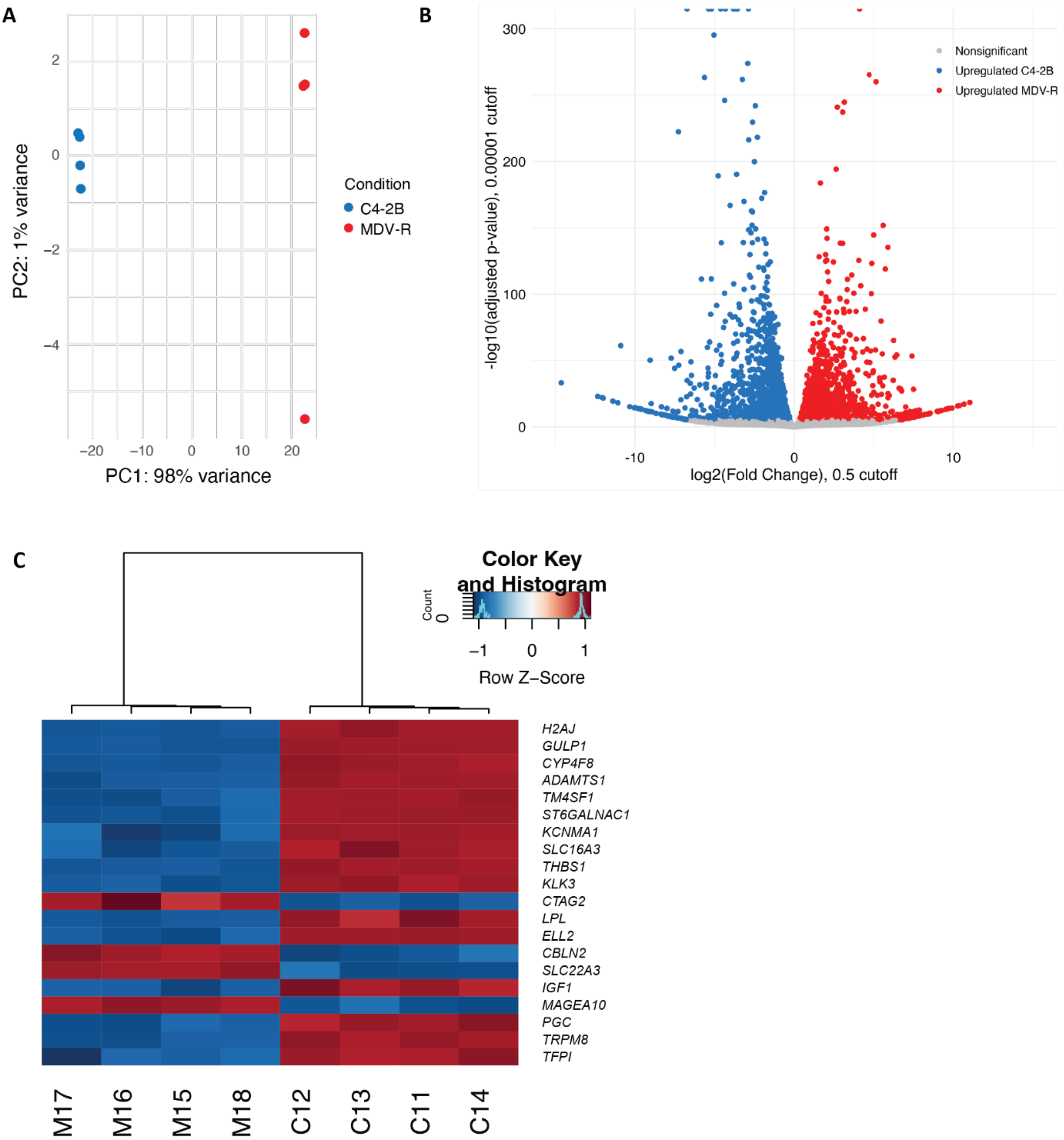

Figure S1. Differential RNA-seq between C4-2B and MDV-R PC cells. (A) PCA plot of RNA-seq samples with 4 replicates per cell line. (B) Volcano Plot of statistically significant differential RNA-seq results (p-adj<0.00001). Downregulated represents the transcripts with log2FC < -0.5 and Upregulated with log2FC > 0.5. (C) Heatmap of top 20 differentially expressed transcripts ranked by log2FC. MDV-R replicates are labeled M5-M8. C4-2B replicates are labeled C1-C4.

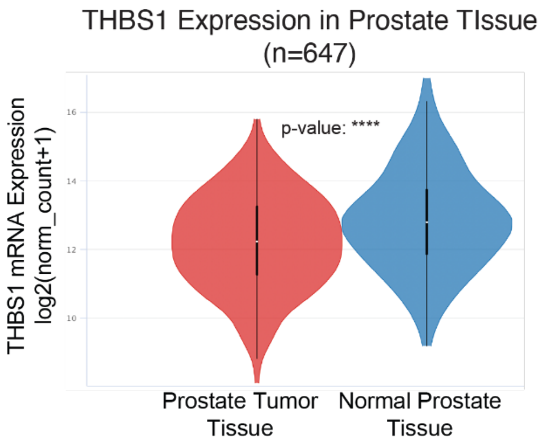

Figure S2. THBS1 expression in normal tissue vs. prostate cancer tissue in PC patients from TCGA. Significance code: **** p < 0.00001.

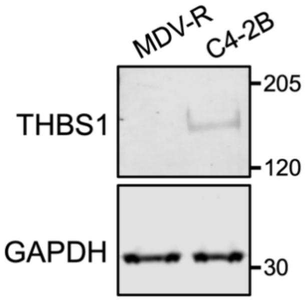

Figure S3. THBS1 protein expression between MDV-R and C4-2B PC cells. Western blot for THBS1 and GAPDH in MDV-R and C4-2B cells.

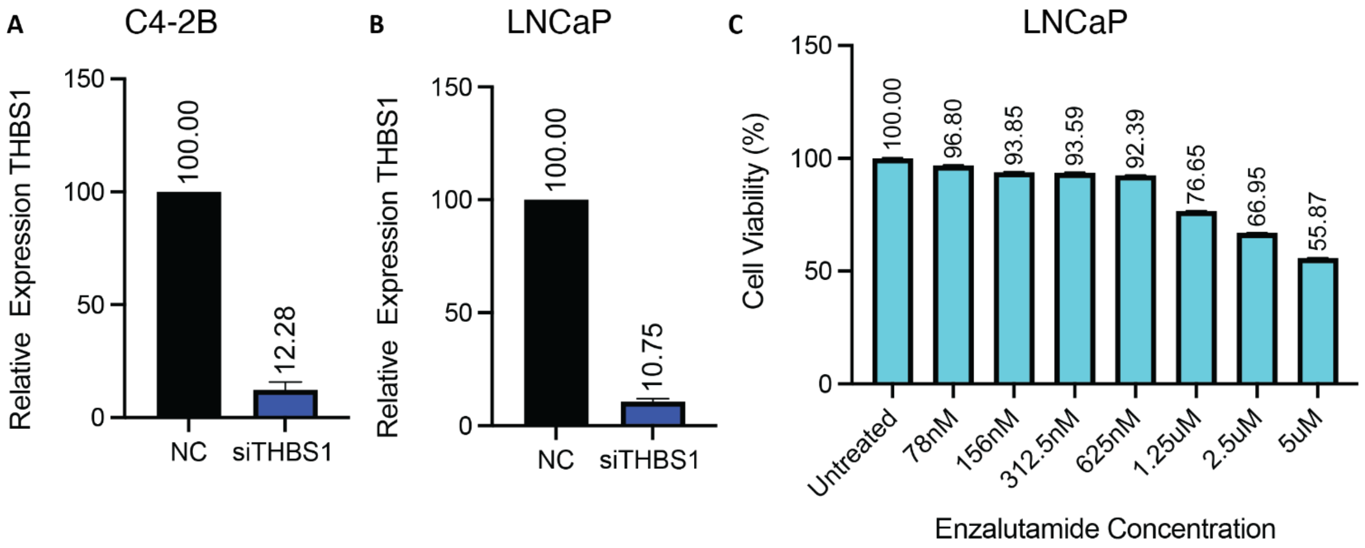

Figure S4. siRNA-knockdown efficiency and enzalutamide viability in PC cells. *THBS1* siRNA-knockdown efficiency for (A) C4-2B and (B) LNCaP cells. NC= negative control (scrambled siRNA). (C) LNCaP cell viability after 72 hours of enzalutamide treatment at varying concentration.

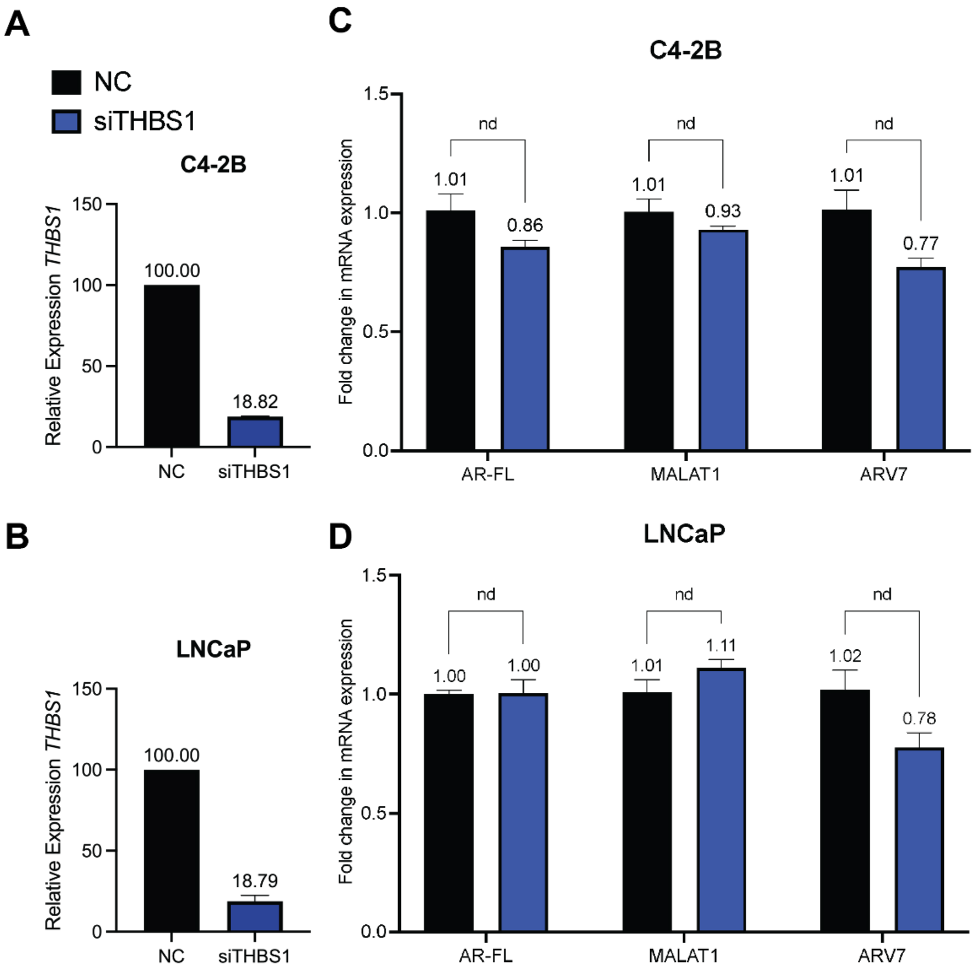

Figure S5. Effect of acute THBS1 depletion on *AR-FL*, *MALAT1*, and *ARV7* expression in PC cells. *THBS1* siRNA-knockdown efficiency for (A) C4-2B and (B) LNCaP cells. NC= negative control (scrambled siRNA). Fold change in mRNA expression of *AR-FL*, *MALAT1* and *ARV7* in (C) C4-2B and (D) LNCaP cells.

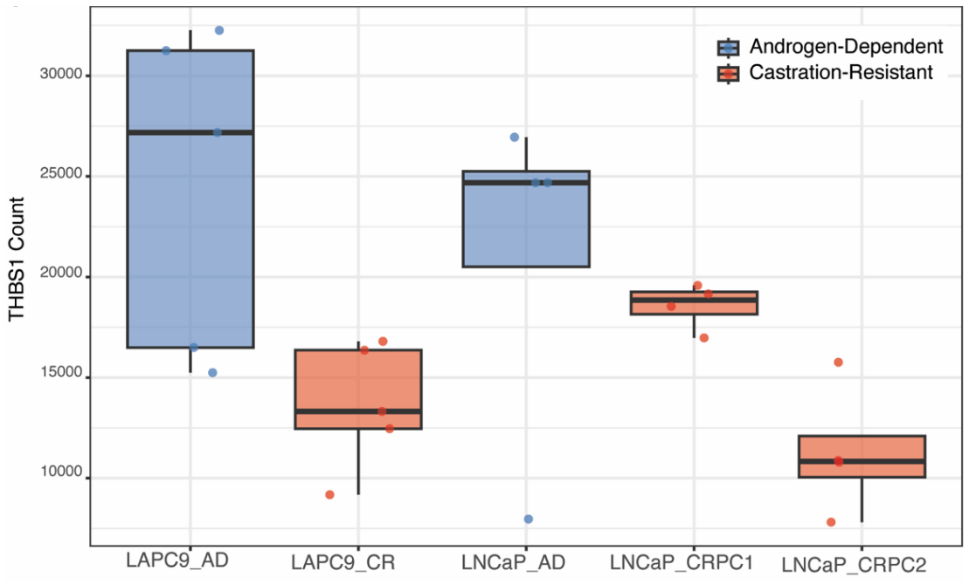

Figure S6. THBS1 Expression in Preclinical datasets before removing outlier from LNCaP_AD replicates. In existing preclinical dataset (PMID: 30190514), THBS1 expression (counts) decreased in androgen-dependent (AD) xenografted LAPC9 and LNCaP PC cell lines after surgical castration of the host. LNCaP_CRPC1 refer to the cell lines derived after surgical castration; LNCaP_CRPC2 refer to the castration-derived cell line after treatment with enzalutamide. P-values presented are from Wilcoxon rank sum test for LAPC9_AD vs LAPC9_CR (p=0.0556), LNCaP_AD vs. LNCaP_CRPC1 (p=0.3429), LNCaP_AD vs. LNCaP_CRPC2 (p=0.2) LNCaP_CRPC1 vs. LNCaP_CRPC2 (p=0.0286).
